# We are not all coping: a cross‐sectional investigation of resilience in the dementia care workforce

**DOI:** 10.1111/hex.12419

**Published:** 2015-10-16

**Authors:** Kate‐Ellen J. Elliott, Christine M. Stirling, Angela J. Martin, Andrew L. Robinson, Jennifer L. Scott

**Affiliations:** ^1^ School of MedicineWicking Dementia Research and Education CentreFaculty of HealthUniversity of TasmaniaHobartTas.Australia; ^2^ School of Health Sciences (Nursing and Midwifery)Faculty of HealthUniversity of TasmaniaHobartTas.Australia; ^3^ The Tasmanian School of Business and EconomicsUniversity of TasmaniaHobartTas.Australia; ^4^ School of Health Sciences (Nursing and Midwifery)Faculty of HealthUniversity of TasmaniaHobartTas.Australia; ^5^ School of Medicine (Psychology)Faculty of HealthUniversity of TasmaniaHobartTas.Australia

**Keywords:** community, dementia, distress, formal care, resilience, workforce

## Abstract

**Background:**

Research on workforce development for high‐quality dementia care more often focuses on enhancing employee knowledge and skill and less on managing employee stress and coping at work.

**Objective:**

To review employee stress and coping in response to high job demands in community‐based dementia care organizations in Tasmania, Australia.

**Methods:**

Stress and coping in response to job roles of 25 community‐based dementia care workers were reviewed using self‐report questionnaire data. Data were analysed for descriptive results and at an individual case level. Individual participant scores were reviewed for clinically significant stress and coping factors to create *worker profiles of adjustment*.

**Results:**

Two adjustment profiles were found. The *‘global resilience’* profile, where workers showed positive adjustment and resilience indicating they found their jobs highly rewarding, were very confident in their abilities at work and had a strong match between their personal and organizational values. The second *‘isolated distress’* profile was only found in a minority and included poor opportunities for job advancement, a missmatch in personal and work values or clinically high levels of psychological distress.

**Conclusion:**

Aged care workplaces that advocate employee well‐being and support employees to cope with their work roles may be more likely to retain motivated and committed staff. Future research should consider employee stress and coping at the workforce level, and how this can influence high‐quality care delivery by applying the measures identified for this study. Comparative research across different care settings using meta‐analytic studies may then be possible.

## Introduction

Dementia is a debilitating neurodegenerative disease which impacts on every health and social care system in the world.^1^ There are grave concerns that the current aged and dementia care workforce is not prepared to manage the exponential growth of dementia. By 2050, it is estimated there will be 981 000 people in Australia with dementia, trebling current numbers (256 500).^2^ While a threefold increase in care workers is required to meet this demand,^3^ several other elements of health services influence the adequacy of our health‐care systems to surmount this challenge. Low levels of recruitment and retention of employees in the aged care sector^4^, ^5^ and low qualifications^6^ hinder quality care for people with dementia.^7^, ^8^ Low recruitment and retention of staff in aged and dementia care not only impacts on the quality of care, it is associated with high costs in a low‐resource setting.^9^ Problems with the stability of the workforce mean there are limited opportunities for consistency of care delivery, which is vital for people with dementia. This combination of circumstances means current health services policies are not meeting the complex and varied needs of the workforce and the aged and dementia populations they serve.^10^, ^11^

The heterogeneous nature of dementia has many implications for the workforce and service providers operating within aged care. In fact, within the older adult group, Pachana and Helmes^12^ suggested that there is probably no other patient population that presents such diversity for health professionals than people diagnosed with a form of dementia. Understanding how workers react to undertaking this care role for people with dementia is vital, particularly to determine whether stress and coping play a role in staff decisions to stay or leave their employers, and further how this affects care delivered to people with dementia. In this paper, we refer to *stress and coping* as an interactive process. *Stress* is the psychological and physical response to a situation that is challenging to endure, whereas *coping* is the strategy or approach an individual applies to manage and endure the stress response or symptoms associated with the situation. Stress and coping factors greatly influence a person's well‐being and psychological adjustment to engage effectively with usual activities in life including work. Research on the well‐being of dementia care workers mostly applies to the residential care setting and not the community‐based arena.^13^, ^14^

Understanding the stress and coping of the dementia care workforce is of particular concern due to the connection between job stress and turnover.^15^ Aged care workers in America leave their jobs because they are dissatisfied at being unable to provide quality care.^16^ General health status may also influence worker commitment. Poor self‐rated health was found in a higher proportion of Australian aged care workers who intended to leave their jobs within the year than workers who intended to stay.^5^ While 13 drivers of retention, intention to stay and intention to leave have been identified in the Australian aged care workforce,^5^ a focus on both stress and coping of workers has to our knowledge not previously been explored. Howe and colleagues^5^ suggested future research should explore factors that address instability in the workforce, rather than retention efforts. Understanding the role of stress and coping in the stability of the dementia care workforce will inform policies that target turnover and quality care for people with dementia and their families.

There is uncertainty about the stress aged and dementia care workers experience. A systematic review^13^ found a lack of strong evidence for a distressed workforce across studies in 24‐h care settings. Two studies included in the review reported dissimilar prevalence rates of staff distress with Astrom, Nilsson, Norberg and Winblad^17^ finding 37 per cent prevalence, whereas Kuremyr, Kihlgren, Norberg, Astrom and Karlsson^18^ reported 5 per cent of staff were ‘at risk’ from burnout. In contrast, four studies reported low mean stress scores.^13^ Results from studies on the well‐being of the aged and dementia care workforce are at best mixed, particularly in comparison with other workforces who have more consistent and established patterns of stress and coping (i.e. nurses, police and rescue workers^19^, ^20^, ^21^, ^22^). Developing a better understanding of stress and coping for the dementia care workforce is a priority. The consistent use of psychological measures with sound psychometrics will enable better comparison across workplace settings and aid the establishment of normative data to determine the precise nature, and patterns of stress and coping in this occupational group. This is of particular relevance considering aged and dementia care workers are an important occupational group for the future of dementia care. Identifying and adopting the use of measures commonly adopted in the clinical psychology setting which have been applied to other workforces may offer some solution to this problem.

The most difficult aspects of working with people with dementia include coping with, and managing behavioural and psychological symptoms of dementia (including aggression and hostility), particularly when care workers are not trained to manage these situations. The resistive, difficult and unpredictable nature of these behaviours were found to be a challenge for Australian nurses working in residential care.^23^ While overall, most nurses were satisfied with their jobs, a quarter reported that working with people with dementia did not provide any job satisfaction.^23^ For dementia care workers, job satisfaction is multifaceted and these workers can experience satisfaction in their job tasks and with the organization, while also experiencing negative perceptions of the clients they work with. While Brodaty *et al*.^23^ examined job strain and satisfaction for dementia care workers, the frequency of positive and negative emotional responses at work (such as feeling excited or upset) was not assessed. Including a review of workers positive and negative emotions at work may help to identify stress and coping patterns that influence workers decisions to stay or leave their workplaces.

Aged and dementia care workers who stay in their jobs longer experience less stress at work. While early career long‐term (residential) care workers in America experienced stress more than later career workers (employed more than 2 years), they also experienced more hopeful and person‐centred attitudes about people with dementia.^14^ Length of experience in care work seems to affect the way workers perceive their jobs, in particular early career workers express overall higher levels of psychological arousal (i.e. presence of both distress and hope). It is possible that being a carer for a long period of time reduces care workers’ level of intense reaction to job situations and events. With more experience, care workers may develop coping strategies that reflect resilience to habituate to on‐going work demands. Including a review of workers positive emotions (e.g. excited, enthusiastic, inspired^24^) may help to identify stress and coping patterns that include a focus on resilience and positive adjustment to working with people with dementia and whether these apply at all stages of workers careers.

Social psychological theory offers a lens for this focus on stress and coping for the aged and dementia care workforce. Self‐efficacy, the belief in one's own ability to successfully accomplish or perform a specific behaviour,^25^ can be a strong indicator of resilience. In the clinical psychology literature, self‐efficacy or confidence to cope predicts better adjustment to a range of chronic stressors.^26^ Applying a clinical psychological approach will help to further understand stress and coping factors essential to managing the demands of providing care to people with dementia. Occupational self‐efficacy or confidence in abilities to cope at work may be an essential element of worker longevity and the stability of the dementia care workforce into the future.

The work environment is an essential part of stress and coping for employees. The greater the mismatch between a person's values and the organization's values, the more distress the person experiences.^27^, ^28^ This is referred to person‐organization fit,^27^ which has not previously been examined in the aged and dementia care workforce and was investigated in our study. When a work environment has resources available to deliver specialized care, employees experience lower job demands, less job strain and distress when exposed to care recipients with disruptive behaviours, than those employees where such resources are unavailable.^29^ Employees who work in a care environment not optimized for care delivery may experience distress easily relieved by changes to their work environment. Community‐based care workers, who deliver support in the home of the person with dementia,^30^ may experience distress that is influenced by the resources available in their work environment.

This study aimed to deepen understanding about stress and coping of aged and dementia care workers based in the community. The effects of stress on workers, associated with the dementia care role, were investigated using psychometrically sound measures. Specific stress and coping areas were assessed that have theoretical links to workforce resilience. Results will be discussed in the context of occupational stress literature in dementia care (mostly focused on residential care) to review if similarities exist for the community setting.

## Method

### Setting

This study formed part of the Work 4 Dementia Project^30^ and was conducted with community‐based care organizations providing support services to people with dementia. Organizations were Home and Community Care (HACC) funded services from Tasmania, Australia. HACC funds basic maintenance and support services to help frail older people and younger people with disabilities to continue living in their community. Employees were invited to participate if they provided care and assistance to people with dementia and worked in the community care environment. Employees were eligible to participate if they delivered some form of care assistance or support to a person with dementia either (i) in the care recipient's home or (ii) completed home visits as part of service delivery or (iii) performed tasks that enable the care recipient to remain in the community, such as shopping or (iv) a combination of these tasks. Employees working in residential care facilities were not included in the study.

### Design

The Work 4 Dementia Project aimed to investigate ways to build capacity and resilience for the dementia care workforce and included several interrelated studies. The study reported here used a cross‐sectional survey design using known constructs of workforce resilience. This method was chosen to further investigate qualitative findings from a previous study within the project^30^ about employee experiences in dementia care. Twenty‐five participants were recruited following convenience sampling from HACC services in Tasmania, Australia. Recruitment procedures were the same as the quali‐tative study, and participants were recruited over a 6‐month period (April to October 2010). Forty workers registered interest in the project, but eight of these could not be contacted and three did not meet study inclusion criteria. Of the 29 eligible participants, three refused to participate due moving house, undergoing surgery and incorrect expectations of the study. One participant did not attend the interview and withdrew.^31^ The data reported in this paper were collected from participants’ answers on a set of self‐report questionnaires. Participants completed a booklet containing the questionnaires during their own time, in private, away from their work site. Questionnaires assessed stress and coping domains (see Table 1). Ethics approval was granted in February 2010 (EC00337: reference number H10984).

**Table 1:** Summary of quantitative self‐report measures for the Work 4 Dementia Project

Measure reliability (Chronbach's α)	Description participants asked to rate…	Range of scoring high score = *Clinical cut‐off score*	Normative comparison sample Mean (SD)
Psychological distress Kessler 10 (K10; 0.71^52^, ^53^)	… the frequency of negative emotional states in the last month using a five‐point Likert scale (1 = none of the time to 5 = all of the time). (e.g. ‘about how often did you feel so nervous that nothing could calm you down’)	0–50 = high risk of mental disorder 10 to 15 Low 16 to 29 Medium 30 to 50 High	Community population aged 55–65 years^54^ *N* = 8841 13.9 (0.2)
Positive and Negative Affect Schedule (PANAS^55^), (+ ve 0.89/−ve 0.85^24^)	… rate frequency of positive and negative emotions on a five‐point Likert scale (0 = none at all, 4 = extremely). (e.g. ‘excited/upset’)	0–50 PA = positive adjustment NA = depression	General population^24^ *N* = 1003 31.31 (7.56) 16.00 (5.90)
Satisfaction with Life Scale (SWLS; 0.80 to 0.89^56^)	… extent of agreement on seven‐point scale (1 = strongly disagree to 7 = strongly agree). (e.g. ‘In most ways my life is close to ideal’)	0–30 = high life satisfaction *above 25 satisfied with life*	Health workers^57^ *N* = 225 23.6 (6.1)
Occupational Self‐Efficacy Scale (OSES; 0.92^58^)	… ability to cope with work challenges on a six‐point Likert scale (1 = completely true, 6 = not at all true). (e.g. ‘As far as my job is concerned I am a rather self‐reliant person’)	20–120 = low levels of occupational self‐efficacy	University students^58^ *N* = 153 19.99 (6.10)
Job Satisfaction Survey (JSS; 0.60–0.82 subscales and 0.91 total score^59^)	… level of satisfaction with their job on various areas (i.e. salary, promotion, supervision, fringe benefits, contingent rewards, operating procedures, co‐workers, work and communication) using a six‐point Likert scale, (1 = disagree very much, 6 = agree very much). (e.g. ‘I like doing the things I do at work’)	36–216 = highly satisfied *above 144 satisfied with job*	Health workers^59^ *N* = 3148 133.1 (27.9)
Organisational Commitment Questionnaire (OCQ^60^), (0.89^61^)	… their level of commitment to the organization on a seven‐point scale (1 = strongly disagree – 7 = strongly agree). (e.g., ‘It would take very little change in my present circumstances to cause me to leave this organization’‐ R)	0–72 = high intent to stay	aAbove the 75th percentile 54
Subjective Person‐Organisation Fit Scale (SPOF; 0.88^62^)	… feelings of congruence between personal and organizational values on a five‐point Likert scale (1 = strongly disagree to 5 = strongly agree).(e.g. ‘I feel that my personal values are a good fit with this organization’)	4–16 = high congruence	aAbove the 75th percentile 12.25
Alzheimer's Disease Knowledge Test^63^ (ADKT; 07.1 ‐ 0.92;^64^)	… level of dementia knowledge using multiple choice responses.(e.g. ‘Although the rate of progression of Alzheimer's disease is variable, the average life expectancy after onset is: (a) 6 months–1 year (b) 1–5 years (c) 6–12 years (d) 15–20 years (e) I don't know’)	0–17 = good knowledge about AD	Patients, carers and non‐carer adults^65^ 50% correct

a When normative comparisons unavailable then the cutoff score was set at the 75th percentile; R = item reverse scored.

### Data analysis

Stage 1 of the analysis included descriptive statistics at the group level. Stage 2 of the analysis uniquely applied a case‐based clinical psychology approach, similar to pattern analysis. This method is used frequently in the clinical psychology and psychiatry literature to describe adjustment to a chronic stressor (i.e. medical condition^32^, ^33^) and has been applied to the occupational health field.^34^, ^35^ This approach also enables comparisons between the current sample and general and clinical populations on indices of coping and mental health. While these comparative samples were not always similar in setting (e.g. students), the norms are not available elsewhere. This is the first study to apply these measures to this occupational group. To compensate for the limitations of the normative comparison sample, a stringent approach for clinically recognized cut‐off scores (usually 1 Standard Deviation) was used (we applied 2 SDs) to find patterns outside the ‘normal response’, which could include those participants ‘at risk’ of developing clinical disorders as identified by established clinical parameters. We applied 2 SDs to improve validity that participants would not fall within the ‘normal range’ on stress and coping measures. For example, a score above 27.8 (2 SDs away from the mean of the normative sample) on the Negative Affect Scale is associated with a depressive disorder.^24^ Stage 2 of the analysis adopted in this study follows the first part of a method used by Jacobson and Truax^36^ to determine whether treatment gains were made after psychological therapy. A similar approach has been applied using a small sample where subgroups (e.g. profiles) were determined by focusing on individual scores against clinical cut‐offs.^37^ Following these approaches, this study used a comparison method that evaluates participant scores to those of others (normative sample) with a focus on criteria that identify a departure from dysfunctional sample or potential to meet a psychiatric diagnosis.^38^

### Self‐report measures

The main stress and coping indices (see Table 1) used to assess participants included psychological distress, frequency of positive and negative emotions, satisfaction with life, confidence in abilities at work, job satisfaction, person‐organization fit, knowledge of dementia and intention to stay at work. The measures adopted to assess these areas have been used extensively in the occupational health psychology literature. The measures have published normative data, which enables examination of participants’ functioning in the context of relevant occupational or clinical samples, and good reliability and validity. Coefficient alphas ranged from 0.71 (Kessler 10) to 0.95 (Occupational Self‐Efficacy Scale). This enabled a more extensive analysis focused on case‐based presentations.

Questionnaire data were entered into IBM SPSS Statistics 19.0.^39^ Raw data were converted and tallied using Syntax to create total scores for all measures. At the group level, data were analysed to produce descriptive statistics for the sample. During the case‐based analysis, individual participant scores were reviewed for clinically significant adjustment (e.g. at least two standard deviations away from the mean normative comparison sample or clinical cut‐off) across intrapersonal and occupational domains to create *worker profiles of adjustment*. The overall pattern of scores across each domain for each participant was reviewed. Cases were then grouped together based on similarities between scores on each measure. Finally, comparisons were made to create *profiles* based on scores on the measures, as well as personal background and professional characteristics.

## Results

Participants were community dementia care workers (*n* = 25) with an average age of 53 years (SD = 9.6). The majority were women (88%) and employed on a casual basis (64%). Most were employed on average for 6 years (SD = 4.3) and on average worked 25.9 h per week (SD = 10.2). Participants were mostly (*n* = 11) married (44%) and earned over (*n* = 9) $65 000 income annually from all sources (35%). Most workers (*n* = 22) received an orientation to their work on commencement of their jobs (88%). These results are comparable to a recent Australian survey on the aged care workforce which indicated community‐based care workers were mostly aged between 45 and 54 (37.2%) or 55 and 64 (29.7%) years, and worked between 16 and 34 h per week (56.4%). Table 2 shows a comparison of the current sample characteristics to the Australian community‐based workforce.

**Table 2:** Sample characteristics in comparison to Australian survey results

	Work 4 dementia (*N* = 25)	Australian survey (*N* = 5214)
	*N* (%)	%
Sex		
Men	3 (12)	10
Women	22 (88)	90
Employment basis		
Permanent full‐time	0 (0)	7
Permanent part‐time	9 (36)	63
Casual	16 (64)	30
Hours worked per week		
1–15	5 (20)	19
16–34	12 (48)	56
35–40	8 (32)	20
>40	0 (0)	5
Employed in one job	16 (64)	86
Employed in more than one job	9 (36)	14
Years in current job		
1 or less	3 (12)	15
2–4	7 (28)	35
5–9	5 (20)	31
10+	9 (36)	19
Country of birth		
Australia	20 (80)	72
England	4 (16)	8^a^
Malaysia	1 (4)	–
Qualification type		
Certificate II	3 (12)	–
Certificate III	11 (44)	59^a^
Certificate IV	8 (32)	17^a^
Degree	3 (12)	–
Received past professional psychological support	6 (24)	13–20^a^/8^a^
Attended past training in dementia	13 (52)	48

Table 1 adapted from Elliott *et al*. (2013) by including data on community‐based direct care workers from The Aged Care Workforce Report.^40^

a Differences in the survey items: ^1^includes South Africa and Ireland; ^2^aged care qualifications only; ^3^self‐rated work illness due to stress or mental health condition vs. organizational reports of workers with illness due to stress or mental health condition; ^4^self‐rated health as ‘fair’ or ‘poor’.

### Descriptives

On average most participants were well adapted, but the range in scores shows some participants were not coping (see Table 3). These results were clinically significant with scores two standard deviations away from the normative comparison showing positive adjustment in confidence in abilities at work, person‐organization fit and opportunities for job promotion. Two outcomes (approached significance), dissatisfaction with supervision, and experience of positive emotions at work (±1 SD). Overall, there were no mean scores on any measure that indicated clinically significant poor functioning in any of the assessed areas. Data were reviewed to look for patterns as to why some participants were coping well, while others were not. When individual cases were reviewed, they fell into one of two profiles.

**Table 3:** Descriptives for community‐based dementia care workers

Measures	Range		$\overset{¯}{x}$	SD	Qualitative description
	Min	Max			
Psychological distress, Kessler 10	10	24	14.72	3.78	Likely to be mentally well
Positive and negative affect schedule					
Positive subscale	30	50	39.80^+1SD^	5.51	High positive emotions at work
Negative subscale	10	23	14.24	3.88	Normal negative emotions at work
Satisfaction with life scale	6	30	20.12	6.65	Generally satisfied – like some improvement
Occupational self‐efficacy scale	28	59	42.44^a^	8.46	Very high confidence in work ability
Job satisfaction survey	129	174	154.92	12.60	Overall satisfied with job
Pay subscale	14	22	18.64	2.23	Normal satisfaction with pay
Promotions subscale	19	23	21.52^a^	1.42	Highly satisfied with promotion
Supervision subscale	14	19	15.20^−1SD^	1.50	Dissatisfied with supervision
Benefits subscale	14	22	18.48	2.22	Normal satisfaction with benefits
Contingent rewards subscale	14	21	17.08^a^	2.33	Highly satisfied with rewards
Operating procedures subscale	9	20	14.24	3.29	Normal satisfaction with operation
Co‐workers subscale	14	22	16.96	2.11	Normal satisfaction with co‐workers
Nature of work subscale	19	22	19.36	0.81	Normal satisfaction with nature of work
Communication subscale	9	19	13.44	3.19	Normal satisfaction with communication
Organisational commitment questionnaire	15	79	53.31	13.11	Normal intent to stay
Subjective person‐organisation fit scale	4	16	12.37^a^	3.92	Very high values match with organization
Knowledge of Alzheimer's disease scale	5	13	9.20	2.24	Low knowledge in dementia (54% correct)

^±^^1SD^: one standard deviation above or below the mean of the normative sample comparison.

a Clinically significant adaptive function (±2 SDs above or below the mean of the normative sample comparison).

### Individual profiles of worker stress and coping

We categorized worker's scores on all self‐report measures by *caseness*, firstly indicating adaptive functioning and secondly indicating poor functioning to form *global resilient* and *isolated distress* profiles of adjustment. Global resilient adjustment was considered to include workers who showed an absence of clinically significant levels (e.g. at least two standard deviations away from the normative mean for a comparative sample or clinical cut‐off scores) of poor functioning as measured by the questionnaires on areas of psychological well‐being. In addition, workers with global resilient profiles showed clinically significant adjustment on various occupational areas and or psychological well‐being. The *isolated distress* profile included dysfunction in job demands and or psychological well‐being areas, whereas global resilient profiles showed adaptive function in these areas. While workers in the isolated distress profile showed clinically significant levels of poor adjustment in occupational and or well‐being domains, some workers showed positive adjustment in other areas (e.g. satisfaction with life or positive affect). The term ‘isolated distress’ is therefore used to describe this profile to acknowledge this pattern of scores.

### Global resilience profile

Overall, 64 per cent of workers showed resilient profiles of stress and coping as measured by the assessed areas. These employees found their jobs rewarding, including being satisfied with opportunities for promotion (94 per cent showed clinically high levels of satisfaction with opportunities for promotion at work). Eighty‐eight per cent of resilient workers were confident in their abilities at work indicated by clinically high levels of occupational self‐efficacy. They also felt their personal values matched those of the organizations (81 per cent reported clinically high congruence with organizational values) and were committed to working for the organization into the future (56 per cent of scores on organizational commitment were clinically high). Fifty per cent of employees in this profile reported high satisfaction with supervision from a leader in their organization. Twenty‐five per cent had high knowledge of Alzheimer's disease, and 18 per cent showed high satisfaction with pay. Only one worker reported high levels of positive emotions at work demonstrated by scores on positive affect.

Workers scoring positively on occupational resources and well‐being were more likely to be in a committed relationship than not, and more likely to have worked for over 5 years, and were currently working above 20 h per week. Most resilient workers had a household income of more than $35 000, Certificate III or above qualifications and had attended training in dementia.

### Isolated distress profile

Overall, 36 per cent of workers were of interest, as they scored outside the typical resilient profile. Key elements that stood out in this group were high psychological distress, poor match with organizational values, life dissatisfaction and poor opportunities for promotion, as well as low commitment to the organization. Thirty‐three per cent of workers in the isolated distress profile had clinically significantly poor psychological adjustment, as assessed by the K10. In fact, in clinical terms, these workers scored in the *likely to have a mild mental disorder* range, meaning they were experiencing significant psychological distress. Thirty‐three per cent of workers in the isolated distress profile did not hold the same values as their organization indicated by clinically significantly low scores of congruence with their workplace values. Another 33 per cent of individuals were dissatisfied with life (one of these workers, also had significant psychological distress and poor match with the values of their organization). Two workers reported significantly low levels of opportunities to advance their job, and one worker reported not being committed to the organization in the future indicated by significantly low score on organizational commitment. In the isolated distress profile, 66 per cent of workers had poor knowledge of dementia evidenced by answering less than half of the questions on the assessment of Alzheimer's disease knowledge correctly; however, this was not significantly different from the normative population.

Workers scoring poorly on psychological well‐being were not in a committed relationship (divorced, never married or widowed), employed on a casual basis and had not undertaken training in dementia. These workers were more likely to be women, have Certificate III or below as highest level of education, have household incomes below $35 000 and had employment for <5 years with their current employer. The individual reporting low organizational commitment (i.e. high intention to leave) also met similar criteria, however, had worked for 10 years with their employer and held a degree and a Certificate IV. Overall, workers with poor function on occupational domains and well‐being were more likely to hold Certificate III or above, did not completed training in dementia and were not in a committed relationship.

## Discussion

Overall, dementia care workers reported positive function, but some cases showed workers with isolated distress. On average, community dementia care workers showed high occupational self‐efficacy, high job satisfaction in contingent work rewards and promotional opportunities, as well as high congruence with organizational values. Similar results were found for an Australian national survey showing community‐based workers were satisfied with their jobs.^40^ Although not significant, two trends in this study showed workers’ experienced positive affect, but they were dissatisfied with supervision. An evaluation of caseness showed two distinct adjustment profiles characterized by scores on psychological measures that reflected (i) global resilience and (ii) isolated distress.

Global resilience profiles showed positive adjustment across a range of occupational and intrapersonal domains to suggest that resilient workers were extremely confident in their work ability, believed their personal values matched those of their employer and found their jobs satisfying and rewarding. In comparison, employees with an isolated distress profile faired much worse, as job resources were low (poor job advancement opportunities and miss‐match in personal and work values), and for some, levels of psychological distress were high (enough to warrant psychological treatment). Demographic and occupational differences between profiles showed disparity across workers. Resilient workers were more likely to have longer length of employment (5 years and above), be in a committed relationship, hold higher levels of education and qualifications, earn higher incomes and have dementia‐specific training. These findings are consistent with a World Health Organisation^41^ report into mental health and resilience, which suggest factors important for well‐being and mental health include educational attainment, social cohesion and quality relationships, as well as stable employment and financial security. The implications of the findings from the current study align with recommendations from the WHO report, which suggest employment opportunities and workplaces that promote and protect mental health through organizational policies and practices are a priority for action to improve the resilience of the dementia care workforce internationally. Future research may focus on workplace settings that addresses the acceptability and feasibility^42^ of stress and coping intervention programmes for dementia care employees.

Career status (in terms of length of employment) influenced how workers experienced distress. Later career workers were more likely to identify with a resilient rather than an isolated distress profile, which is supported by findings from Zimmerman *et al*.^14^ These results highlight the importance of orientation programmes in the sector, particularly at the beginning stages of employment. Confidence in job tasks during early career stages may also play an important role for workforce resilience. This study showed high occupational self‐efficacy (e.g. confidence in job tasks) was associated with global resilience profiles. In the clinical psychology literature, individuals with high confidence to cope with chronic stressors have better adjustment than those with low confidence.^26^ Applying theoretical concepts essential for adjustment and coping in the clinical psychology literature to the occupational setting for aged care may widen the understanding of workforce resilience and capacity building for dementia care.

While there was no direct finding of high organizational support, results suggested leadership through supervision, a type of organizational support, may be more important for workers with isolated distress than those with global resilience profiles. Research on residential aged care workers in Australia supports this finding, as supervisor support interacts with job demands to show that when job demands are either too low or too high, well‐being and satisfaction are negatively impacted.^11^ Guidance and mentorship may be a greater need for workers with isolated distress to help alleviate burdens associated with decision‐making and problem‐solving as part of the paid care role. Leadership in this workforce often involves supervision, where workers are able to seek advice and support that extends to include debriefing and social support from a more senior work colleague. Therefore, organizational support in the form of collegial interaction and guidance (via supervision) appears to play a role in the adjustment of community‐based dementia care workers. This is supported by previous research^31^ suggesting social interaction at work in the form of *occupational communion* may have adaptive benefits for workers’ coping and adjustment. In addition, Hartling^43^ argued that resilience can be strengthened through relationships that foster personal growth, which has implications for the workforce. An organization that fosters supportive professional relationships that lead to personal growth may have a workforce capable of adjusting to high job demands.

Limitations of the study include the generalizability of the findings. While this sample was similar on most national community‐based characteristics including mostly female, born in Australia, worked between 16 and 34 h per week, approximately half attended training in dementia, and approximately one‐fifth with a possible mental illness,^40^ a greater proportion were casually employed, Certificate IV trained and had been employed with their organization for 10 years or more. Higher rates of casual employment might be symptomatic of the challenges with resourcing an island state and other regional areas. Such geographic locations are vulnerable to workforce shortage, lending to higher rates of casual and part‐time employment.^44^ The difference in length of time with employer might be due to the tendency for Tasmanian workers to relocated less frequently (e.g. interstate) than other Australian states.^45^ This could partly explain why participants remained with their employers for longer than the national average. Further, the number one reason given by Australian community‐based workers for leaving their position was due to a change in location.^40^ These issues do not occur in isolation in the Australian aged and dementia care setting, as much research has indicated similarities internationally.^4^

The percentage of workers in this study with poor psychological well‐being matched that of the general population, as 20 per cent of Australian adults experience psychological problems such as mental illness in any year.^46^ In our study, the origins of stress were unknown, and assessing whether the isolated distress experience related to workplace or existing mental health conditions was not evaluated. There is, however, clear evidence in the literature of bidirectionality in the relationship between psychological distress and work related factors. Cohort longi‐tudinal studies and multilevel studies show that employee group agreement about work conditions, predicts employees’ mental health^47^). Despite the limitation of the cross‐sectional design of this study, the group processes that occur in a workplace when a small proportion of employees are clearly ‘not coping well’ remains an area for future investigation. For example, it may be likely that employees with isolated distress profiles leave the workplace as their sense of belonging at work is undermined alongside a majority of resilient employees. Future research through replication studies is needed to closely examine a larger number of the workforce that experience distress in the community‐based dementia care setting. Investigations may focus on whether high levels of employee distress effect service delivery and quality dementia care. Despite using psychometrically sound measures, some of the main findings were based on subscales (e.g. satisfaction with opportunities for job promotion and finding work rewarding) which can be less reliable than total scores and can increase the likelihood of type II errors.

A strength of the study is the use of well validated and reliable psychometric tools to assess work psychological function. Applying the case‐based clinical psychology approach enabled the comparison of dementia care workers function to clinical and normative samples. This approach is distinctive within dementia care research. However, some caution is warranted due to the differences between this sample and the normative comparison samples. Overall, dementia care workers showed good fit with the values and goals of community‐based services and similar findings have been found in other types of effective organizations.^48^ This study offered an insight as to the preparedness of workers to cope with high demands. Those workers likely to experience distress in the face of high demands may be those less engaged with organizations values and future directions. Raising education levels and qualifications in the sector may also improve resiliency of the workforce by having strong knowledge and self‐efficacy that will contribute to preparedness.

Workforce resilience has previously been explored in several occupational groups such as child protection services,^49^ psychotherapists treating trauma patients^50^ and general nursing.^51^ Commonly the notion of dealing with adversity by ‘bouncing back’ is central to the resilient experience in all work settings. In the community‐based dementia care setting, most employees adjust well to the demands of the job, which include coping with strong emotional responses such as grief and loss at work.^31^ For the minority of dementia care workers, who experience high distress, access to employee assistance programmes and an organization that promotes and protects mental health is a high priority. Our study offers a way forward to investigate the stress and coping of the dementia care workforce. If our study only reported results based on a group mean, then it would indicate that no intervention is required, but this commonly adopted approach would have missed the one‐third of employees who were not coping. More research is required to build normative data on stress and coping in this setting. Health policymakers and aged care organizations may consider the stress and coping factors highlighted by this study, including supervision, education and training, opportunities to improve self‐efficacy, and person‐organization fit, as areas to focus on in order to sustain and build a resilient workforce.

## Sources of funding

This work was supported in part by a grant from the Tasmanian Home and Community Care Program (HACC), a jointly funded State and Australian Government program awarded to Scott et al. (grant number S0017329). This research was also supported in part by a PhD scholarship jointly provided by TIME for Dementia (Tasmania and Victoria Dementia Training Study Centre, Australia), the Wicking Dementia Research and Education Centre and the University of Tasmania, Australia (045135, awarded to J.L.S and A.L.R; K.J.E stipend).
